# Hyperuricemia and risk of increased arterial stiffness in healthy women based on health screening in Korean population

**DOI:** 10.1371/journal.pone.0180406

**Published:** 2017-06-30

**Authors:** Hoon Young Choi, Seok-hyung Kim, Ah Ran Choi, Seung Gyu Kim, Hyunwook Kim, Jung Eun Lee, Hyung Jong Kim, Hyeong Cheon Park

**Affiliations:** 1 Department of Internal Medicine, Gangnam Severance Hospital, Yonsei University College of Medicine, Seoul, Korea; 2 Severance Institute for Vascular and Metabolic Research, Yonsei University College of Medicine, Seoul, Korea; 3 Department of Internal Medicine, Yongin Severance Hospital, Yonsei University College of Medicine, Seoul, Korea; 4 Department of Internal Medicine, CHA Bundang Medical Center, CHA University, Seongnam, Korea; Universita degli Studi di Milano, ITALY

## Abstract

Hyperuricemia is a risk factor for cardiovascular disease and is associated with increased arterial stiffness in high-risk populations. However, given the possible sex-related differences in the prevalence of hyperuricemia, the association between elevated serum uric acid (SUA) level and increased arterial stiffness has yielded conflicting results. We investigated the relationship between SUA and arterial stiffness in asymptomatic healthy subjects who underwent a health examination. Subjects who underwent a comprehensive health examination were enrolled. After exclusion of extensive confounding factors, 2,704 healthy subjects with coronary calcium score < 100 were evaluated in the final analysis. All subjects underwent brachial—ankle pulse wave velocity (baPWV) to detect arterial stiffness. The SUA was divided into quartiles for its association with arterial stiffness and was analyzed separately for men and women. The mean SUA level was significantly lower in women than in men. The baPWV was significantly elevated in subjects with the highest quartile of SUA in women, but not in men. After adjusting for age, smoking, systolic blood pressure, body mass index, estimated glomerular filtration rate, fasting plasma glucose, high-density lipoprotein-cholesterol, low-density lipoprotein-cholesterol, and coronary artery calcium score, the highest quartile of SUA in women was significantly associated with increased risk of high baPWV compared with the lowest quartile of SUA (OR = 1.7, *p* = 0.018), whereas in men, SUA level was not associated with high baPWV. Our study showed that elevated SUA is independently associated with increased baPWV in healthy Korean women, but not in men.

## Introduction

Elevated serum uric acid (SUA) levels are a common finding in patients with hypertension, metabolic syndrome, and renal disease [1]. Studies in populations of patients at high risk for cardiovascular disease (CVD) have shown an association between high SUA levels and CVD [2]. Furthermore, in patients with moderate-to-severe chronic heart failure, high SUA levels increase all-cause mortality independent of other risk factors [3], and this increase in risk seems to start at an SUA level above 7 mg/dL [4]. Even in relatively healthy populations, high SUA levels are significantly associated with increased subclinical coronary atherosclerosis [5]. Interestingly, a sex-dependent association, particularly in women, has been suggested between higher SUA level and the risk of adverse cardiovascular (CV) events [6, 7].

Arterial stiffness is one of the earliest signs of adverse structural and functional changes within the vessel wall. It causes an increase in the conductance velocity of an impulse along the vessel that can be measured by pulse wave velocity (PWV). Brachial—ankle pulse wave velocity (baPWV) is a noninvasive and simple method to determine arterial stiffness [8]. Several studies have shown that increased baPWV is an independent predictor of CV morbidity and mortality [9].

The detrimental effect of hyperuricemia on arterial stiffness was previously reported in some cohort studies and meta-analyses [10, 11]. A cohort study of the general population in Korean rural communities also reported hyperuricemia as an independent risk factor for arterial stiffness [11] However, confounding factors, such as underlying hypertension, diabetes mellitus, chronic kidney disease, gout, coronary artery disease, and medications, which could have influenced the development of arterial stiffness, were not excluded in many of the studies [12–14]. Moreover, a cohort study from Okinawa and a large meta-analysis found no predictive role for uric acid in coronary heart disease and considered the uric acid as only a component involved in the clustering of traditional CVD risk factors [15, 16]. Given these discordant results, the association between hyperuricemia and arterial stiffness remains controversial. In addition, this association has been relatively underexplored in healthy subjects. To investigate the role of uric acid per se in arterial stiffness, enrolling subjects without traditional CVD risks would be preferable. The present study enrolled only asymptomatic individuals who underwent extensive cardiac risk factor evaluation and had coronary artery calcium score (CACS) of less than 100. Therefore, this study aimed to determine the relationship between SUA level and baPWV in healthy men and women subjects, considering the multiple confounding factors, including CACS.

## Materials and methods

### Study population

**Fig 1:**
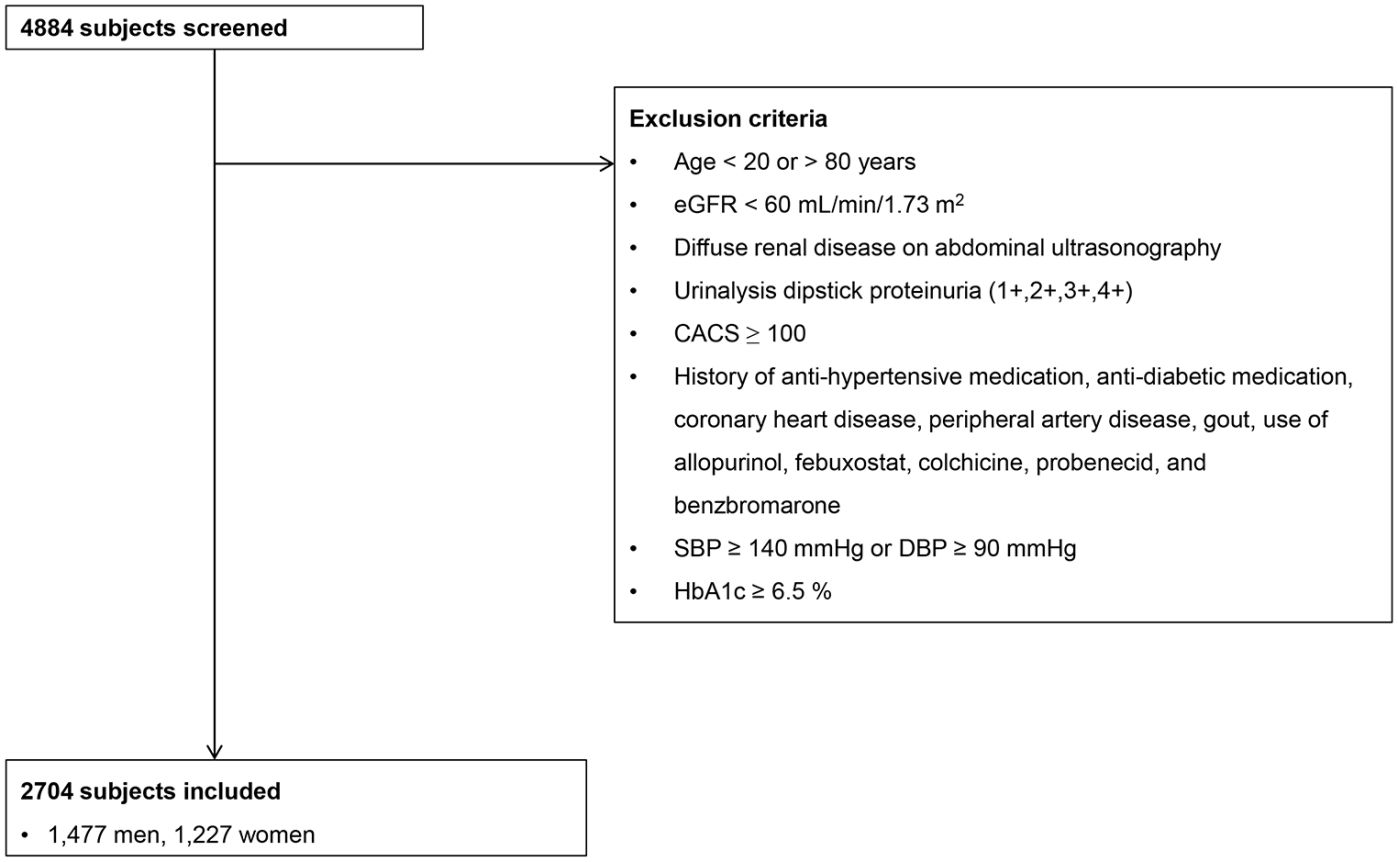
Study design.

In this cross-sectional single-center study, we consecutively collected data for 4,884 subjects who underwent baPWV and cardiac multi-detector computed tomography (MDCT) as part of a comprehensive health examination at Gangnam Severance Hospital, South Korea, from July 2006 to September 2013. Our study only included participants aged between 20 and 80 years. To eliminate the influence of confounding variables, subjects with a history of hypertension, diabetes mellitus, coronary artery disease, peripheral artery disease, or gout, and those taking medications, such as allopurinol, febuxostat, colchicine, probenecid, and benzbromarone, were excluded. A history of medical illness including gout was ruled out through intensive questionnaires and consultations with doctors. Subjects who had evidence of chronic kidney disease (CKD) were also excluded. The definition of CKD was based on the presence of kidney damage (dipstick proteinuria of 1+, 2+, 3+, or 4+), decreased kidney function (eGFR < 60 mL/min/1.73 m^2^), or diffuse renal disease on abdominal ultrasound. Furthermore, subjects with measured systolic blood pressure (SBP) ≥ 140 mmHg and diastolic blood pressure (DBP) ≥ 90 mmHg, CACS ≥ 100, or glycosylated hemoglobin (HbA1c) ≥ 6.5% were excluded. Finally, 2,704 subjects (1,477 men, 1,227 women) were included in this study (Fig 1). The Institutional Review Board of Gangnam Severance Hospital, Yonsei University College of Medicine, approved the study protocol (No. 3-2015-0128), and informed consent was waived owing to the retrospective nature of the study.

### Measurement of clinical variables and confounding variables

We reviewed the patients’ medical records, including history of hypertension, diabetes mellitus, and smoking status. Height, body weight, and blood pressure were measured during hospital visits. Blood samples were obtained on the day of the MDCT scan, after a fasting period of at least 12 hours. The following laboratory parameters were determined: hemoglobin, calcium, phosphorus, blood urea nitrogen (BUN), creatinine, fasting glucose, total cholesterol, triglyceride, low-density lipoprotein (LDL)-cholesterol, high-density lipoprotein (HDL)-cholesterol, and uric acid. The SUA levels were divided into sex-specific quartiles as follows: in men, Q1: 113.1–309.4, Q2: 309.41–351.1, Q3: 351.11–398.7, and Q4: 398.71–719.9 μmol/L; in women, Q1: 119.0–226.1, Q2: 226.1–255.9, Q3: 255.91–291.6, and Q4: 291.61–517.7 μmol/L. Kidney function was determined based on estimated glomerular filtration rate (eGFR). eGFR was calculated using the formula developed and validated in the Modification of Diet in Renal Disease (MDRD) study [17] as follows: eGFR = 186 × serum creatinine^−1.154^ × age^−0.203^ (× 0.742, for women).

Proteinuria was determined by urine dipstick analysis and reported semiquantitatively as negative, trace, 1+, 2+, 3+, or 4+. A result recorded as negative or trace was defined as the absence of overt proteinuria. Body mass index (BMI) was calculated as body weight (kilograms) divided by the square of height (square meters). Subjects were classified as current smoker or non-smoker based on smoking habit; non-smokers included those who had never smoked and ex-smokers.

### PWV measurement

baPWV was measured with a volume plethysmography device (VP-1000, PWV/ABI; Colin Co., Komaki, Japan) after the subjects had rested for at least 5 min. This automated device records the phonocardiogram, electrocardiogram, volume pulse form, and arterial blood pressure at the left and right brachial arteries and ankles. The baPWV was calculated using time-phase analysis between the right brachial artery pressure and the volume waveforms at both ankles. The distance between the brachium and the ankle was calculated automatically according to the subject’s height. After measurement of the right and left baPWV, we used the mean baPWV as a marker of arterial stiffness. The validity, reliability, and reproducibility of this equipment were demonstrated according to previous study methods [18]. High baPWV was defined as a value ≥1,400 cm/s [9, 19].

### MDCT

Subjects were scanned with a cardiac MDCT system (Philips Brilliance 64; Philips Medical Systems, Best, The Netherlands) with 3-mm slice thickness and 1.5-mm reconstruction interval. With the subject in the supine position, cardiac MDCT was performed in the craniocaudal direction within a single breath-hold at end-inspiratory suspension. Subjects with an initial heart rate > 66 beats/min before cardiac MDCT examination received a β-blocker (25 mg atenolol; Tenormin, Hyundai, Seoul, Korea), unless β-adrenergic blocking agents were contraindicated. Iodinated contrast agent (Optiray 350; Tyco Healthcare, Kantata, Canada), at a dose of 2.0 mL/kg not exceeding a total of 100 mL, was administered using a two-phase injection protocol for the arterial and delayed phases of the CT images. Quantitative calcium scores were calculated according to the method previously described by Agatston et al. [20].

### Statistical analyses

All analyses were performed using the Statistical Package for the Social Sciences (SPSS, version 20; Chicago, IL, USA). The data are expressed as mean ± standard deviation or standard error for continuous variables or as percentages for categorical variables. Differences between continuous and categorical variables were analyzed using the independent *t*-test and the chi-squared test, respectively. Univariate logistic regression analyses were used to derive relationships between high baPWV and clinical variables. To identify independent factors predicting high baPWV, we used multivariate logistic regression analysis to determine odds ratios (OR) and 95% confidence intervals (CI). We adjusted for traditional cardiovascular risk factors including age, SBP, BMI, eGFR, fasting plasma glucose, HDL-cholesterol, LDL-cholesterol, current smoker, and CACS based on the univariate logistic regression analysis. SUA levels were divided into sex-specific quartiles. The mean baPWV of each quartile was analyzed using ANCOVA (analysis of covariance), considering the confounding factors. In a post-hoc subgroup analysis, patients in the age group with cardiovascular risk were categorized as “younger” and “older” (male ≥ 45 years, female ≥ 55 years) [21]. A *p*-value < 0.05 was considered statistically significant.

## Results

### Characteristics of study population

The average age of the study population was 50.6 ± 8.9 years (men, 50.4 ± 8.7 years; women, 50.9 ± 9.1 years), and 54.6% of the participants were male. The mean SUA level was 309.4 ± 77.9 μmol/L, which was significantly lower in women than in men (260.0 ± 54.1 μmol/L vs. 353.4 ± 68.4 μmol/L, *p* < 0.001). Apart from age, all the other parameters—proportion of current smokers, SBP, DBP, BMI, eGFR, levels of BUN, creatinine, fasting plasma glucose, SUA, total cholesterol, triglycerides, HDL-cholesterol, and LDL-cholesterol, CACS, and mean baPWV differed between men and women (Table 1). Given the difference in their mean SUA level, we performed the rest of the analysis separately for men and women.

**Table 1:** Clinical characteristics of the study population by sex.

Clinical variables	Men (n = 1477)	Women (n = 1227)	*p*-value
Age (years)	50.4 ± 8.70	50.9 ± 9.1	0.146
Smoking n (%)	289 (19.6)	34 (2.8)	<0.001
SBP (mmHg)	121 ± 10	115 ± 12	<0.001
DBP (mmHg)	77 ± 7	72 ± 8	<0.001
BMI (kg/m^2^)	24.3 ± 2.7	22.0 ± 2.7	<0.001
eGFR (mL/min/1.73 m^2^)	92.5 ± 21.2	98.17 ± 28.5	<0.001
Blood urea nitrogen (mmol/L)	5.0 ± 1.1	4.7 ± 1.2	<0.001
Creatinine (μmol/L)	88.4 ± 17.7	61.9 ± 17.7	<0.001
Fasting plasma glucose (mmol/L)	5.3 ± 0.7	5.0 ± 0.6	<0.001
Uric acid (μmol/L)	353.4 ± 68.4	260.0 ± 54.1	<0.001
Total cholesterol (mmol/L)	5.0 ± 0.9	5.1 ± 0.9	<0.001
Triglycerides (mmol/L)	1.5 ± 0.9	1.0 ± 0.5	<0.001
HDL-cholesterol (mmol/L)	1.2 ± 0.3	1.5 ± 0.3	<0.001
LDL-cholesterol(mmol/L)	3.1 ± 0.8	3.2 ± 0.8	0.032
CACS	6.16 ± 17.42	1.87 ± 8.80	<0.001
Mean baPWV (cm/s)	1333.6 ± 157.3	1297.2 ± 187.8	<0.001

*Note*: Data are means ± standard deviation and number (percentage) of subjects.

Abbreviations: SBP, systolic blood pressure; DBP, diastolic blood pressure; BMI, body mass index; eGFR, estimated glomerular filtration rate; HDL, high-density lipoprotein; LDL, low-density lipoprotein; CACS, coronary artery calcium score; baPWV, brachial—ankle pulse wave velocity.

The clinical characteristics of men and women according to normal and high baPWV are shown in Table 2. The percentage of subjects with high baPWV (≥1,400 cm/s) was 26.3% (n = 710). Significant differences were found in age, SBP, DBP, BMI, eGFR, and CACS between the normal and high baPWV groups for both sexes. However, fasting plasma glucose, SUA, total cholesterol, triglyceride, HDL-cholesterol, and LDL-cholesterol levels differed significantly between subjects with normal baPWV and those with high baPWV only in women, but not in men (Table 2).

**Table 2:** Comparison of the clinical characteristics between normal baPWV subjects and high baPWV subjects by sex.

Clinical variables			
**Men (n = 1477)**	**baPWV < 1400 (cm/s) (n = 1053)**	**baPWV ≥ 1400 (cm/s) (n = 424)**	***p*-value**
Age (years)	48.9 ± 8.2	54.1 ± 8.9	<0.001
Smoking n (%)	186 (17.7)	103 (24.3)	0.010
SBP (mmHg)	112 ± 11	125 ± 9	<0.001
DBP (mmHg)	76 ± 7.1	79 ± 6	<0.001
BMI (kg/m^2^)	24.5 ± 2.8	23.8 ± 2.5	<0.001
eGFR (mL/min/1.73 m^2^)	93.3 ± 21.7	90.4 ± 19.8	0.017
Fasting plasma glucose (mmol/L)	5.3 ± 0.7	5.4 ± 0.9	0.149
Uric acid (μmol/L)	357.0 ± 65.5	351.1 ± 71.4	0.157
Total cholesterol (mmol/L)	5.0 ± 0.9	5.0 ± 0.9	0.940
Triglycerides (mmol/L)	1.5 ± 0.9	1.5 ± 0.8	0.637
HDL-cholesterol (mmol/L)	1.2 ± 0.3	1.2 ± 0.3	0.167
LDL-cholesterol (mmol/L)	3.1 ± 0.8	3.1 ± 0.9	0.508
CACS	5.1 ± 15.3	8.9 ± 21.5	<0.001
Mean PWV (cm/s)	1259.0 ± 89.6	1519.0 ± 134.7	<0.001
**Women (n = 1227)**	**baPWV < 1400 (cm/s) (n = 941)**	**baPWV ≥ 1400 (cm/s) (n = 286)**	***p*-value**
Age (years)	48.9 ± 8.5	57.4 ± 7.9	<0.001
Smoking n (%)	26 (2.7)	8 (2.7)	0.908
SBP (mmHg)	113 ± 12	123 ± 10.1	<0.001
DBP (mmHg)	70 ± 8	77 ± 7	<0.001
BMI (kg/m^2^)	21.8 ± 2.6	22.6 ± 2.7	<0.001
eGFR (mL/min/1.73 m^2^)	99.28 ± 28.2	94.5 ± 29.1	0.013
Fasting plasma glucose (mmol/L)	4.9 ± 0.5	5.1 ± 0.6	<0.001
Uric acid (μmol/L)	255.9 ± 53.6	273.7 ± 59.5	<0.001
Total cholesterol (mmol/L)	5.0 ± 0.9	5.3 ± 1.0	<0.001
Triglycerides (mmol/L)	0.9 ± 0.4	1.1 ± 0.6	<0.001
HDL-cholesterol (mmol/L)	1.5 ± 0.3	1.4 ± 0.3	<0.001
LDL-cholesterol (mmol/L)	3.1 ± 0.8	3.4 ± 0.9	<0.001
CACS	1.2 ± 7.1	4.0 ± 12.7	<0.001
Mean PWV (cm/s)	1219.2 ± 110.9	1553.8 ± 158.3	<0.001

*Note*: Data are means ± standard deviation and number (percentage) of subjects.

Abbreviations: SBP, systolic blood pressure; DBP, diastolic blood pressure; BMI, body mass index; eGFR, estimated glomerular filtration rate; HDL, high-density lipoprotein; LDL, low-density lipoprotein; CACS, coronary artery calcium score; baPWV, brachial—ankle pulse wave velocity.

**Fig 2:**
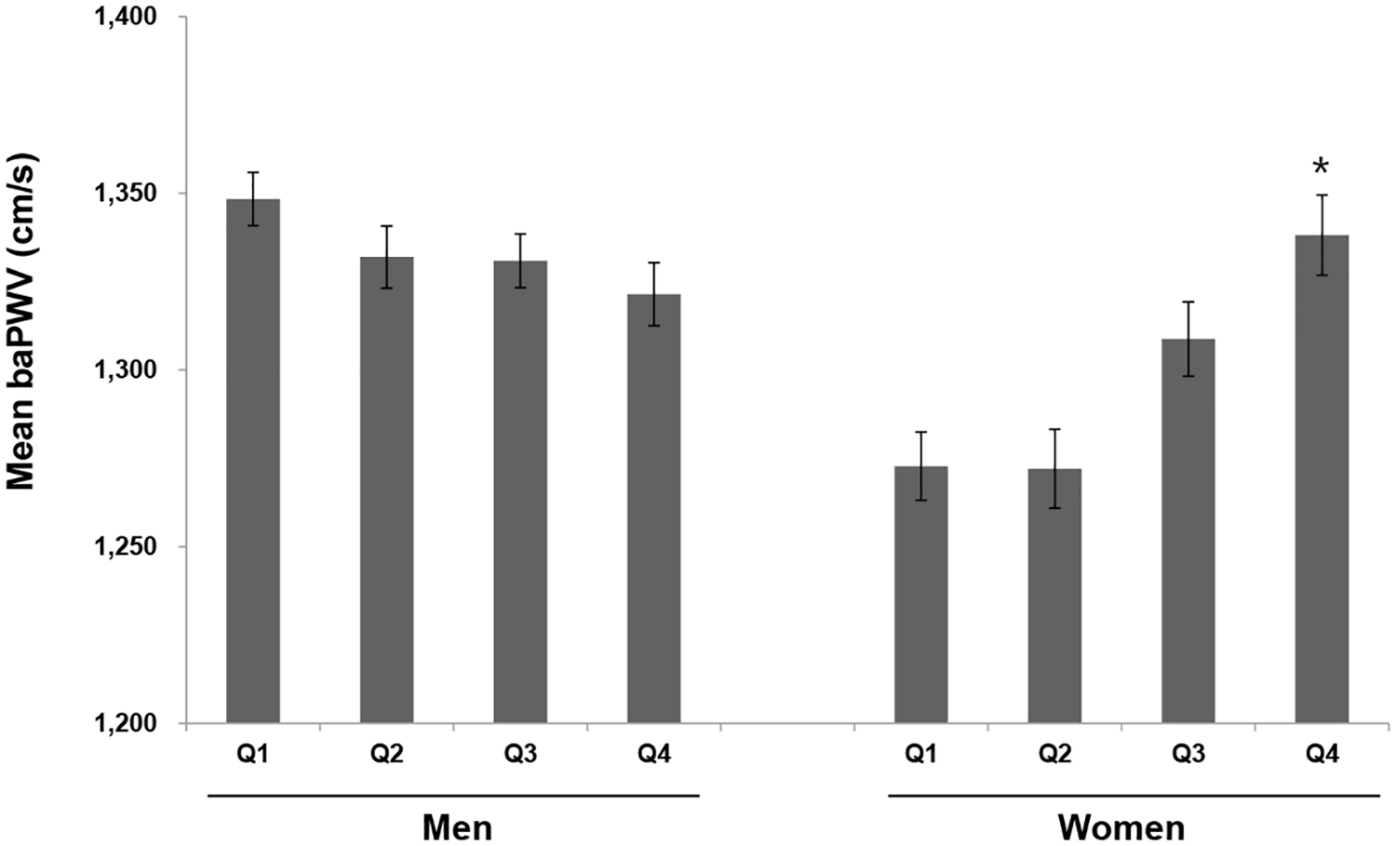
Sex-based differences in baPWV by serum uric acid level. Comparison of baPWV among subjects with serum uric acid quartiles by sex, based on ANCOVA with adjustment for age, smoking, SBP, BMI, fasting glucose, HDL-cholesterol, and LDL-cholesterol. The SUA levels were divided into sex-specific quartiles as follows: in men, Q1: 113.1–309.4, Q2: 309.41–351.1, Q3: 351.11–398.7, and Q4: 398.71–719.9 μmol/L; in women, Q1: 119.0–226.1, Q2: 226.1–255.9, Q3: 255.91–291.6, and Q4: 291.61–517.7 μmol/L. *p < 0.05 compared with Q1 of SUA.

Based on ANCOVA with adjustment for age, smoking, SBP, BMI, and level of fasting glucose, HDL-cholesterol, and LDL-cholesterol, baPWV in the highest quartile of SUA levels was significantly increased compared with that in the lowest quartile of SUA in women, whereas in men, baPWV in each quartile of SUA was not different (Fig 2).

### Relationship between SUA and arterial stiffness in each sex

Univariate logistic analysis showed that age, SBP, BMI, eGFR, CACS, and levels of fasting plasma glucose, HDL-cholesterol, and LDL-cholesterol correlated significantly with high baPWV in women. An increase of SUA level was associated with a significant increase in the risk of high baPWV in women, whereas SUA level was not significantly associated with high baPWV in men (S1 Table).

In multiple logistic regression analysis, higher baPWV was used as a dependent variable, and SUA or each quartile of SUA was entered as an independent variable (Table 3). After adjusting for age, smoking, SBP, BMI, eGFR, CACS, and levels of fasting plasma glucose, HDL-cholesterol, and LDL-cholesterol, women in the highest quartile of SUA level were associated with increased risk of high baPWV compared with those in the lowest quartile (OR = 1.7, 95% CI 1.1–2.7; *p* = 0.018). By contrast, SUA level was not independently associated with high baPWV in men (Table 3).

**Table 3:** Multivariate logistic regression analysis for high baPWV.

Variables	Men (n = 1477)		Women (n = 1227)	
	OR (95% CI)	*p* value	OR (95% CI)	*p* value
Age	1.1 (1.059–1.094)	<0.001	1.1 (1.096–1.147)	<0.001
Smoking	1.7 (1.222–2.236)	0.001	1.5 (0.545–3.929)	0.450
SBP	1.1 (1.051–1.079)	<0.001	1.1 (1.062–1.095)	<0.001
BMI	0.9 (0.822–0.914)	<0.001	0.9 (0.822–0.940)	<0.001
eGFR	1.0 (0.986–0.998)	0.014	1.0 (0.988–0.999)	0.027
Fasting glucose	1.0 (0.994–1.012)	0.545	1.0 (0.996–1.027)	0.161
HDL-cholesterol	1.0 (0.990–1.014)	0.743	1.0 (0.973–0.998)	0.021
LDL-cholesterol	1.0 (0.990–1.002)	0.312	1.0 (0.997–1.023)	0.353
CACS	1.0 (0.996–1.009)	0.490	1.0 (0.991–1.023)	0.408
Uric acid quartiles				
Q2 vs. Q1	1.0 (0.672–1.334)	0.775	1.2 (0.764–1.984)	0.392
Q3 vs. Q1	1.2 (0.835–1.654)	0.355	1.3 (0.820–1.975)	0.282
Q4 vs. Q1	1.1 (0.755–1.575)	0.646	1.7 (1.099–2.734)	0.018

Abbreviations: SBP, systolic blood pressure; BMI, body mass index; eGFR, estimated glomerular filtration rate; HDL, high-density lipoprotein; LDL, low-density lipoprotein.

Uric acid quartiles: men, Q1: 113.1–309.4, Q2: 309.41–351.1, Q3: 351.11–398.7, and Q4: 398.71–719.9 μmol/L; women, Q1: 119.0–226.1, Q2: 226.1–255.9, Q3: 255.91–291.6, and Q4: 291.61–517.7 μmol/L.

### Subgroup analyses for association of SUA with arterial stiffness in each sex according to age

All participants were grouped by age at risk for CV risk in each sex (men≥45 years, women ≥55 years). Multiple logistic regression analysis demonstrated that the highest quartile of SUA level showed a significant correlation with high baPWV in elderly women (≥55 years) after adjusting for SBP, BMI, eGFR, CACS, and levels of fasting plasma glucose, HDL-cholesterol, LDL-cholesterol (OR = 2.3, 95% CI 1.201–4.514, *p* = 0.012). However, in men older than 45 years, SUA level was not independently associated with high baPWV. Furthermore, in younger participants, SUA level was not significantly associated with high baPWV in both sexes (Table 4).

**Table 4:** Multivariate logistic regression analysis between different quartiles of uric acid and high baPWV in each sex according to age.

Age status^a^				
***Younger age***				
**Uric acid quartiles**	**Men (n = 397)**		**Women (n = 879)**	
	**OR (95% CI)**	***p*-value**	**OR (95% CI)**	***p*-value**
Q2 vs. Q1	0.6 (0.216–1.635)	0.314	0.9 (0.439–1.658)	0.640
Q3 vs. Q1	0.8 (0.326–1.784)	0.763	1.1 (0.578–1.941)	0.852
Q4 vs. Q1	1.3 (0.529–2.995)	0.602	1.3 (0.717–2.519)	0.356
***Older age***				
**Uric acid quartiles**	**Men (n = 1080)**		**Women (n = 348)**	
	**OR (95% CI)**	***p*-value**	**OR (95% CI)**	***p*-value**
Q2 vs. Q1	0.9 (0.644–1.331)	0.676	1.5 (0.765–3.033)	0.231
Q3 vs. Q1	1.2 (0.803–1.666)	0.433	1.3 (0.708–2.545)	0.367
Q4 vs. Q1	0.9 (0.599–1.323)	0.565	2.3 (1.201–4.514)	0.012

^a^Adjusted for smoking, SBP, BMI, eGFR, fasting plasma glucose, HDL-cholesterol, LDL-cholesterol, and CACS. Uric acid quartiles: men, Q1: 113.1–309.4, Q2: 309.41–351.1, Q3: 351.11–398.7, and Q4: 398.71–719.9 μmol/L; women, Q1: 119.0–226.1, Q2: 226.1–255.9, Q3: 255.91–291.6, and Q4: 291.61–517.7 μmol/L.

## Discussion

The results in this study showed that high normal or greater SUA (Q4: 291.61–517.7 μmol/L) was associated with increased baPWV in healthy Korean women. Previous observational data included subjects with risk factors for increased arterial stiffness. To our knowledge, this study is the first to carefully examine the traditional risk factors for CVD and exclude other possible confounding risk factors including coronary calcification to analyze the effects of SUA per se on arterial stiffness.

Among the 4,884 subjects who underwent a health screening program in our hospital, only 2,704 were excluded after the comprehensive assessment including CACS because relatively healthy subjects might have participated in a variety of health screening programs according to their needs. Adjustment for these confounding risk factors and final multivariate logistic analysis showed that the risk of high baPWV in the highest quartile of uric acid was significantly higher than that in the lowest quartile in women, after adjusting for confounding factors, but not in men. High-normal and greater SUA, which is a risk factor for increased arterial stiffness in elderly women who are relatively healthy, should be monitored closely in a clinical setting. Since the risk of CVD increases in postmenopausal women, the high-normal and greater SUA level may further play an important role in CVD. Several studies have reported a relationship between increased SUA level and baPWV in healthy subjects. Chen et al. [13] showed that uric acid is positively associated with aortic stiffness and pressure in Chinese men. However, they failed to exclude confounding factors and included subjects with hypertension or diabetes mellitus, as well as those taking medication. Fang et al. [6] demonstrated that high-normal or greater SUA level is associated with increased arterial stiffness in healthy women, after adjustment for confounding variables. Barbieri et al. [22] further showed that higher uric acid levels are significantly associated with higher prevalence of severe coronary artery disease only in women. Their findings are similar to ours, and this sex-linked difference was also found in other larger studies, such as the Chicago Heart Association Detection Project [23], National Health and Nutrition Examination Survey I epidemiologic follow-up study [24], post hoc analysis of the Losartan Intervention For Endpoint reduction in hypertension trials [25], and Scottish Heart Health Extended Cohort study [26]. The Scottish Heart Health Extended Cohort study demonstrated that uric acid level is more strongly associated with mortality in women than in men [26]. Similarly, a higher internal carotid artery resistive index [27] and an increased risk of silent brain infarction [28] have been reported in women, but not in men. This closer association between SUA level and CV risk factors in women than in men may be related to estrogen effects in women [29–31]. In men, SUA elevation frequently begins at puberty, whereas hyperuricemia is usually delayed until after menopause in women. Estrogen in premenopausal women increases renal clearance of uric acid by inhibiting renal proximal tubular urate reabsorption by organic anion transporters [32]. Women are more susceptible to SUA-associated vascular injury than men are [27, 28]. The subgroup analysis in the present study demonstrated that higher SUA level was significantly associated with high baPWV after excluding other confounding factors in healthy elderly women in this study. However, this association between baPWV and SUA by age was not found in men in all age groups and women aged < 55 years. Furthermore, most women aged more than 55 years were diagnosed as being menopausal (data not shown) using the baseline health examination questionnaire. These findings were similar to a previous report that indicated that SUA is independently associated with arterial stiffness in postmenopausal women [33, 34]. However, the precise mechanism of this association remains to be elucidated.

Several potential mechanisms have been proposed by which uric acid could affect arterial stiffness. SUA may promote vascular smooth muscle proliferation, cause oxidation of LDL [35], upregulate the expression of platelet-derived growth factor [36], and stimulate the inflammatory pathways [37, 38]. In addition, uric acid may exacerbate the progression of arterial stiffness by producing reactive oxygen species via the xanthine oxidase pathway [39]. Hyperuricemia may cause endothelial dysfunction by impairing the generation of nitric oxide in vascular endothelial cells [40], and is associated with coronary calcification in relation to crystal deposition [41].

baPWV is well known to increase with age [42], hypertension [43], diabetes mellitus [44], and CKD [45]. Therefore, we excluded these confounding factors that can increase baPWV. A study design that controlled for renal function, past medical history (hypertension, diabetes mellitus), and medications (uric acid-lowering agents) was adopted. Careful attention was also given to the definition of healthy subjects by excluding those with proteinuria of 1+ or more on dipstick urine analysis.

In this study, the CACS was measured in all subjects by using cardiac MDCT. Data from Multi-Ethnic Study of Atherosclerosis demonstrated that addition of CACS to a prediction model based on traditional CV risk factors significantly improves risk classification [46]. Accordingly, utilizing CACS should increase the accuracy of detecting subclinical atherosclerosis in asymptomatic health examination participants and therefore screen subjects with high CV risks. A CACS of more than 100 has been proposed to indicate the presence of atherosclerosis [47]. Thus, we excluded subjects with CACS greater than 100 to establish strict inclusion criteria for enrolling healthy subjects.

Age and increased blood pressure were independently associated with increased arterial stiffness in the present study, and these results are in line with those of previous studies [12, 19, 42, 48, 49]. Our study showed that hyperglycemia was associated with a higher risk of high baPWV in women, but not in men. This result is consistent with previous findings of a greater association between insulin resistance and arterial stiffness in women than in men [50].

baPWV ≥ 1,400 cm/s is used as an indicator of increased arterial stiffness [9]. This cutoff value corresponds to a moderate Framingham risk score and represents the threshold at which the risk for incident hypertension also increases in normotensive individuals. This cutoff value also corresponded to the highest quartile of baPWV for healthy women subjects in the present study.

Our results showed that current smoking was significantly associated with increased arterial stiffness in men, but not in women. This lack of association between smoking habit and increased arterial stiffness in women may be attributed to the small number of women categorized as smokers (n = 34).

This study has several limitations. First, due to its cross-sectional design, we could not demonstrate a causal relationship between SUA level and increased arterial stiffness. Our results need to be further confirmed in a prospective study. Second, we cannot exclude the possibility that our findings were influenced by dietary intake. Certain dietary habits [51], meat or seafood intake, and alcohol consumption are associated with a higher prevalence of hyperuricemia [52]. However, the Korean Multi-Rural Communities Cohort study showed that the dietary intake of their participants failed to show association between dietary factors and baPWV [11]. Third, proteinuria was evaluated with the dipstick method, which is a semiquantitative method that cannot detect microalbuminuria. However, this method has been used widely in general health examination screening, and its usability and reliability have been verified [53]. Finally, we used baPWV instead of carotid-to-femoral PWV (cfPWV) or heart-to-femoral (hfPWV). The prognostic value of baPWV is lower than that of cfPWV, but recent studies have shown that baPWV is associated with both central and peripheral PWVs [54]. It is also useful as a surrogate marker for various CV risk factors, as well as for cfPWV and hfPWV [8, 18, 55–57].

## Conclusion

We found that elevated SUA level is associated with increased arterial stiffness in elderly healthy Korean women. However, the relationship between elevated SUA level and increased arterial stiffness was not significant in men.

## Supporting information

S1 Table Univariate logistic regression analysis between variables and high baPWV. (DOCX)
