# Supplementary material for: Hyperuricemia and risk of increased arterial stiffness in healthy women based on health screening in Korean population
Source: PLoS One. 2017 Jun 30;12(6):e0180406. doi: 10.1371/journal.pone.0180406 (PMC5493403; doi:10.1371/journal.pone.0180406)
Supplement: S1 Table — (DOCX) [file pone.0180406.s001.docx]

**S1_Table. Univariate logistic regression analysis between variables and high baPWV**

| **Variables** | **Men (n = 1477)** | | **Women (n = 1227)** | | |
| --- | --- | --- | --- | --- | --- |
|  | **OR (95% CI)** | ***p* value** | **OR (95% CI)** | ***p* value** | |
| Older age | 2.812 (2.050–3.858) | <0.001 | 5.764 (4.274–7.773) | | <0.001 |
| Smoking | 1.606 (1.185–2.178) | 0.002 | 0.996 (0.426–2.331) | | 0.993 |
| SBP | 1.047 (1.034–1.061) | <0.001 | 1.081 (1.066–1.097) | | <0.001 |
| BMI | 0.921 (0.879–0.966) | 0.001 | 1.102 (1.047–1.160) | | <0.001 |
| eGFR | 0.993 (0.986–0.999) | 0.029 | 0.993 (0.988–0.998) | | 0.011 |
| Fasting glucose | 1.009 (1.001–1.018) | 0.038 | 1.037 (1.022–1.052) | | <0.001 |
| HDL-cholesterol | 1.004 (0.993–1.016) | 0.491 | 0.976 (0.965–0.988) | | <0.001 |
| LDL-cholesterol | 0.998 (0.995–1.002) | 0.420 | 1.009 (1.004–1.013) | | <0.001 |
| Uric acid | 0.928 (0.834–1.033) | 0.173 | 1.434 (1.233–1.667) | | <0.001 |

Abbreviations: SBP, systolic blood pressure; BMI, body mass index; eGFR, estimated glomerular filtration rate; HDL, high-density lipoprotein; LDL, low-density lipoprotein.
